# The Outlook of Medicine and Surgery. No. 4

**Published:** 1883-09

**Authors:** J. McF. Gaston

**Affiliations:** Campinas, S. Paulo, Brazil


### THE OUTLOOK OF MEDICINE AND SURGERY.

Number 4.

By J. McF. Gaston, M.D., Campinas, S. Paulo, Brazil.

Continued from page 669, August Number.

The changes wrought upon the different organs by the use of various electric appliances, through contact with special regions of the body, entitles this therapeutic agency to a more elaborate notice than has been given of other channels of modifying the tissues and constituent elements of the frame of man. The investigative faculty of the far-reaching influence of the multiform application of electricity in many different disorders of the nervous system, and in other derangements of the animal economy places it in the front rank of importance as a curative process, and demands more painstaking observation of the phenomena attending its development, than is generally bestowed upon its use. It approaches as nearly the nature of new power in its transmission from one to another part of the body, that it almost serves as a substitute for the emenations from the nerve centres, and the vital force is stimulated in such form as to regain its vigor in parts that have fallen into an adynamic condition. But the full measure of benefits, confirmed by electricity, extends beyond those affections of the nervous system proper, and are so propagated to other parts as to effect alterations in the secretions of various organs through the nervous distribution to their tissues. The different modes of treating diseases by the constant or interrupted currents of electricity, and by the dynamic and static forms of electricity, have been reduced to the method of science. "One treatment supplementing and re-enforcing the other, results are obtained far more satisfactory than could possibly follow the exclusive use of general or localized faradization, central galvanization or Franklinization.

"The differential indications for the use of galvanism, faradism and Franklinism may well demand the closest scrutiny for the success of our efforts."

Still another mode of influencing the body from without, consists in baths of various temperatures, with fluids or vapors, by which modification of much importance are impressed upon the vital organism. Inhalation of gases and medicated vapors come in also for a share of consideration in recounting the resources for reaching the inner man. This kind of influence is quite distinct from that general result obtained through the inhalation of anæsthetics, which has an extended scope among curative agencies, independent of its employment on a large scale to relieve suffering under surgical operations.

The use of physical exercise, and the absolute rest, with the regulation of diet, pertain to the grand summary means that are available for the connection of the disorders of the human system, and trench upon the department of hygiene, which has for its aim the prevention of diseases in all its forms by obviating developments prejudicial to health.

There remains to be noted the delicate and sensitive operations of the spiritual upon the physical part of the air organization, which is too often overlooked in the treatment of diseases. It is not only in these affections involving to a greater or less extent the emotional faculties, that the mental influence is of consequence to the good issue of a case, but in the ordinary purturbations of the physical elements, the emanations from the mind exert much power upon the performance of the various functions, an agency in the salutary action of medicine which deserves the careful attention of the medical attendant. Faith in the physician adds much to the good effect of the medication prescribed by him.

Having all these channels for the instruction of remedies, the avenues of approach of the diseases are so much more numerous that therapentics cannot boast of great triumphs. It has become generally recognized also in these latter day investigations that the infirmities of the flesh do not depend so much upon natural weakness and decay as upon the ingress of a horde of minute organisms that are seeking to find a suitable nest for propagating their kind in the various tissues of the body. The tree of life is not any longer allowed to rot and wither under the operation of the ordinary laws of disorganization, but a cut worm is at work up its roots, a sawyer strikes its bark, and a caterpillar preys upon its leaves, so that ultimately it falls to the ground a dead mass full of vitality. These destructive tenants abound in all its structure, and the dry rot, which formerly was supposed to play an important part in the gradual decomposition of its fibres, is now attributed to the growth of fungi, spores, microbia and germs of all sorts and signs, that imperceptibly encroach upon its constituent elements. The human body becomes, according to the most approved germ theory of diseases, little else than a hot bed to grow these micro-organisms, and whenever they are depicted in a part favorable to this growth they are rapidly propagated, so as to take on the characteristics of diseased action in the vital structures. The latest nest discerned for these animalculi is in the lungs; and while the eminent experimentalist has satisfied himself that these vermin cause all the trouble in the so-called tubercular consumption, others are so deficient in the tact of hunting small game, as never to have jumped it from its hidden lair, and consequently attribute the ravages in the pulmonary field to some other cause. I

confess, for my own part, that my position is that of the husband high up above the scene of strife between his wife and the bear, when he declared that he had never witnessed a fight in which he felt less concern as to the result, it being a matter of indifference whether tubercular diseases kills the patient through the development of bacilli or by some other species of degeneration in the tissues, and so long as we do not discern a remedy for the mischief the decision of this question can have no practical advantage for physicians.

It has impressed me throughout all this chasing of germs from one part to another, that the simple illustration of the principle of their propagation has been overlooked, and a type of this is afforded in the sore which becomes filled with maggots. It was not the maggots that caused the offensive ulcer, but the wound existing previously; the larvæ of the fly were deposited in it, and found the proper elements for their development and growth. So I am disposed to think that where germs are discovered they are, for the most part, a secondary phenomenon; and at the risk of being considered a doubting Thomas, who must thrust a finger into the wound before accepting the testimony that is adduced in substantiation of this causative element in most disorders, I claim and insist that the proofs are insufficient to establish the germ theory of the origin of disease. That microcosms are propagated in course of the destructive decomposition there can be no question, and my allegation is not against the fact of their existence, but against the claim that they are a causative or constituent element of disease.

Putting aside that immense host of invisible enemies to health as at least of doubtful power in originating disease, we have to deal with many other intangible causes of derangement in the human organism. Indeed there are few endemic or epidemic influences that operate in a way to be subjected to our scrutiny. No one thus far has been able to bottle up malaria and analyze its elementary principles. The decompositions resulting from the decay of vegetable substances effect synthenically a union of particles that produces the miasmatic emanation, but we are at a loss to fix with certainty upon the conditions of its development, and hence unable to lay down established rules for its prevention. It is true that something has been accomplished by the precautionary measures adopted for this end, and it is to be hoped that further investigation will lead to the discovery of the precise origin of atmospheric contamination, so that its pernicious effects may be ultimately obviated. But for the present our task lies in averting, so far as may be possible, the consequences of such poisoning of the blood and secretions, by the antidotes which are contained in the materia medicas at our disposal.

The numerous catarrhal diseases that result from vicissitudes of temperature and varying humidity of the atmosphere, are only recognized by their symptoms, and their corrections have been learned by clinical observation, the treatment being of an empirical character. An analogical proceeding based upon the results of the past, enables the physician to cure a considerable proportion of cases, which if left to the slow process of gradual elimination, would require a much longer period to recover from the disturbances. So it turns out with all medication of diseases, that we determine by what has been done in a given class the remedies to be applied in similar cases, and no general principles have as yet been reached for our guidance in restoring the physical derangements to a normal condition of health.

The desideratum of the practitioner is to ascertain the conditions of healthy development, and the departures from it, so as to adopt uniform measures for correcting these deviations from the normal state; and the medical profession should not rest satisfied with anything short of the solution of this grand and intricate problem. That no single process of treatment can accomplish so desirable a result is very evident, but that means may be discovered to greatly simplify the abnormalities of the human organization, is within the range of probability, and I look forward to the day when comparatively few drugs may be employed to accomplish better results than attained by the extravagant array of medicinal compounds that are now prescribed.

The tendency of most practical observers on the operations of medicines for the cure of diseases is to simplify their prescriptions, and the more experience we have of the mode in which salutary changes are brought about, the less are we inclined to use a great variety of medicines in treating diseases.

The fountain head of energy for all the functions lies in the nerve centres; and by controlling the emanations from this source of power, the vital forces will be propagated with regularity and uniformity to all the remote parts of the physical organization, and it is to this that we must look for the cause of disease.

In like manner it is incumbent upon those who undertake surgical operations to have the nervous system in a favorable state for bearing this insult to the organization, and to adopt measures which may avert the shock or modify the unfavorable impressions resulting from the surgical procedure. The preparation of the system for grave operations is now very generally observed by intelligent surgeons, and the use of anæsthetics in most cases seems to obviate the untoward consequences of pain and of the emotional perturbation. The subsequent employment of tonifying measures with anodynes has also been recognized as essential to the safe management of surgical cases, and there is sufficient ground to believe that the preservation of the vigor of the nerve-centres by such means secures the against the tendency to degeneration of the tissues after extensive operations. It is highly probable that the depressing influence upon the nervous system indicated by shocks may be prevented by a preliminary resort to tonics and stimulants, and a due consideration of the role of the nerve-centres in all vital phenomena should lead the surgeon to anticipate such grave results by timely precautions in this line of prophylactics. As a rule, any derangement of secretions or development of acute disorders is combatted in advance of operating, when the case admits of such delay; and all such procedures have their favorable influences in righting up the performance of the various functions that depend upon the emanations from the nerve centres.

All the animal functions combine in furnishing what is requisite to work this great dynamo machine, upon which depends transmission to all parts of the body; and if its mechanism becomes deranged from any cause there is a vaccilation in the movement or flicking of the light in a portion or the whole of the outposts pertaining to the physical organization. The spinal column may not unaptly be compared to the mechanism of the dynamo that transmits motion, while the ganglionic centres in the great sympathetic correspond to the batteries that propagate the current for electric light, and while each has to be sustained by certain processes, the final result is dependent upon their being kept in good working order. The means then to be adopted for averting injurious impressions upon the nerve centres, and the measures to be used for the correction of their derangements, make up the whole prophylactic combination of hygiene, and include all therapeutic agency in the treatment of diseases, let the details be varied as they may.

The entire armory of the materia medica becomes at last only different steps in attaining the end of correcting the deviations of the nerve centres from the normal performance of their functions. All the emetics, diaphoretics, purgatives, etc., which produce their separate and appropriate influence upon distinct divisions of the complex whole, tend at last to bring about an equilibrium in the action of the various parts of the system; and thus by restoring tone to the several functions, establish the normality of the nerve centres. The nerve force is the *sine qua non* of all vital forces, and it must be the prime consideration of any therapeutic proceeding to reach ultimately the correction of all derangements of the nerve centres.

I am fully aware that this view may be classed with the abandoned theory of vital force, and the consequent resolution of the treatment of disease into stimulants and sedatives; but our concern is not so much with what has been exploded as with that which is sustained by correct observation of facts. There is a well-known proceeding of hunters, when their dogs have overrun the trail, to go back and take up the first scent of the game; and it might not be bad policy in the present headlong chase after novelties to recur to some of the earlier painstaking records of clinical observation, to get upon the right track of the source of the disturbances in the physical organization, and the proper means of correcting them.

The experimentalists of our period are shown to be in error by those of another epoch of later date; and while a furor of approval takes hold of the public mind to day, the condemnatory voice of to-morrow may be heard in in louder tones against a prevalent theory and an existing practice. The good old adage of "all is not gold that shines" has been most strikingly illustrated very recently in regard to a popular haze by which gynecologists were befogged; and we may well take a lesson from such experiences against being led astray by seeming practical results.

While far from ignoring the valuable contributions of scientific physiological and pathological experimenters, it becomes the medical profession to adhere strictly to the cautious records of clinical observation, and thus stay the tendency to enormous deductions from the isolated cases of the laboratory. My appeal is to the law and the testimony for a just decision.

